# Values of procalcitonin and C-reactive proteins in the diagnosis and treatment of chronic obstructive pulmonary disease having concomitant bacterial infection

**DOI:** 10.12669/pjms.333.12554

**Published:** 2017

**Authors:** Yanyan Li, Linlin Xie, Shuzhen Xin, Kaishu Li

**Affiliations:** 1 Yanyan Li, Respiratory Department, Binzhou People’s Hospital, Shandong 256603, China; 2 Linlin Xie, Infection Department (II), Binzhou People’s Hospital, Shandong 256603, China; 3 Shuzhen Xin, ICU, Binzhou People’s Hospital, Shandong 256603, China; 4 Kaishu Li, Respiratory Department, Binzhou People’s Hospital, Shandong 256603, China

**Keywords:** Procalcitonin, C-reactive protein, Acute exacerbations of chronic obstructive pulmonary disease

## Abstract

**Objective:**

To observe the changes in the levels of C-reactive protein (CRP) and procalcitonin (PCT) in serum of patients with acute exacerbations of chronic obstructive pulmonary disease (AECOPD) and to compare with the values of CRP in combination with PCT in the diagnosis and treatment of infective exacerbation of COPD.

**Methods:**

One hundred and sixty-four patients who developed acute exacerbation of COPD and admitted to the Binzhou People’s Hospital from March 2014 to December 2015 were selected. They were divided into an infection group (N=98) and a non-infection group (N=66) according to bacterial culture results of sputum and lung computer tomography (CT) examination results. Moreover, 50 healthy people were selected as a normal control group. The levels of PCT and CRP of the three groups were determined respectively; patients in the infection group and non-infection group were determined again after administration of antibacterial drugs for a period of time. The results were all recorded.

**Results:**

The levels of PCT and CRP of the infection group were significantly higher than those of the non-infection group and the normal control group before treatment, and the difference had statistical significance (P<0.05). The levels of PCT and CRP were (1.97±0.13) μg/L and (7.34±2.66) mg/L respectively in the infection group after treatment, which was much lower than the levels before treatment (P<0.05). The level of PCT of the infection group was remarkably higher than that of the non-infection group after treatment (P<0.05), but the difference of CRP level between the infection group and non-infection group had no statistical significance (P>0.05). The specificity and sensitivity of diagnosing COPD in combination with bacterial infection with PCT or CRP were lower than those of PCT in combination with CRP.

**Conclusion:**

Levels of CRP in combination with PCT is a reliable index for determining the existence of bacterial infection, which is of great clinical guidance significance to the treatment and prognosis assessment of AECOPD patients.

## INTRODUCTION

Chronic Obstructive Pulmonary Disease (COPD) is an incompletely reversible airflow obstruction induced chronic airway inflammation which develops progressively and mainly involves lung.^1^,^2^ The symptoms of patients with acute exacerbations of chronic obstructive pulmonary disease (AECOPD) include cough, short of breath or aggravated wheeze, increased purulent or mucopurulent sputum, etc. The lung function of the patients may be worse continuously, which may even cause death in severe conditions; disease condition can still be aggravated even in remission period.^3^,^4^ Antibiotics are usually used for treating AECOPD in clinics; however, not all AECOPD patients require antibiotic therapy.^5^ Therefore, determining whether patients with AECOPD have bacterial infection rapidly and early is especially important.

The laboratory detection of infectious diseases mainly focused on white blood cell (WBC), erythrocyte sedimentation rate (ESR) and N which were the basis for the application of antibacterial drugs; but the indexes which are lack of specificity may result in misdiagnosis and missed diagnosis, leading to delayed treatment and increased mortality.^6^ C-reactive protein (CRP) and procalcitionin (PCT) are both the resistance products which are generated when pathogenic microorganism invades human body and stimulates cells. In recent years, they have been the new cytokine indexes for diagnosing bacterial infection and have been gradually paid attention to.^7^,^8^ Through observing the changes of PCT and CRP levels, this study investigated the values of them in the diagnosis and treatment of COPD in combination with bacterial infection.

## METHODS

### Instruments and reagents

The level of PCT was detected with Roche Cobas E601 fully automatic electro-chemiluminescence immunoassay analyzer and reagents using electrochemiluminescence. The level of CRP was detected with Roche Cobas 8000 fully automatic biochemical analyzer and reagents using immunoturbidimetry.

### General data

One hundred and sixty-four COPD patients who were admitted to the Binzhou People’s Hospital were randomly selected, including 127 males and 37 females (average age: 66.8±10.2 years). All patients were diagnosed according to The Guidelines of the Diagnosis and Treatment of Chronic Obstructive Pulmonary Disease formulated by COPD team of Respiratory Disease Branch of Chinese Medical Association,^9^ and all of them were at the stage of acute exacerbation, i.e., they had any two of the following symptoms: aggravated anhelation accompanied by gasp and chest distress; aggravated cough and increased purulent sputum; fever; rhonchi and wheezing rale; respiratory failure. The liver function of the patients was featured by incompletely reversible airway limitation. After use of bronchodilators, forced expiratory volume in 1 second (FEV1) < 80% of predicted value and FEV1/forced vital capacity (FVC) < 70%. Patients who had diseases which could induce the increase of PCT and CRP levels, took antibiotics four weeks before admission, required invasive mechanical ventilation, had contraindications to hormones or had disturbance of consciousness were excluded. The enrolled subjects were informed with the purpose and significance of the study. The study has been reviewed and approved by the ethics committee of our Binzhou People’sHospital. All research subjects have signed informed consent. After being diagnosed based on bacterial culture results of sputum and relevant imaging examination results, the patients were divided into an infection group (with positive bacterial culture results) (N=98) and a non-infection group (N=66). In the infection group, there were 79 males and 19 females, with an average age of (66.9±7.1) years. In the non-infection group, there were 48 males and 18 females, with an average age of (67.8±12.6) years. Moreover, 50 healthy people without bacterial infection were selected as a normal control group, including 35 males and 15 females (average age: 67.4±13.2 years). The general data of the subjects in the three groups had no remarkable difference (P>0.05); therefore, the results were comparable.

### Test methods

Firstly, 5ml of fasting venous blood was extracted from each patient using an ordinary biochemical tube which was not added with anticoagulant. After 30 min of still standing at room temperature, the blood was centrifuged at 3000 rpm for 10 min. Then the serum PCT and CRP levels were detected. The PCT and CRP levels of the normal control group were detected in the same conditions. The disease conditions became clinically stable after a period of treatment using antibacterial drugs. Antibacterial drugs were stopped when imaging examination suggested relieved pulmonary inflammation; venous blood was extracted for the detection of PCT and CRP levels in the morning on the second day. The levels of PCT and CRP were compared before and after treatment.

### Statistical methods

SPSS 21.0 was used for data processing. The data were expressed as mean±standard deviation (SD). The differences were analyzed by one-way analysis of variance and t test. Difference was considered as statistically significant if P<0.05.

## RESULTS

### Comparison of serum PCT and CRP levels before treatment

The levels of PCT and CRP of the infection group were higher than those of the non-infection group and the normal control group before treatment, and the difference had statistical significance (P<0.05); the levels of PCT and CRP of the non-infection group were higher than those of the normal control group before treatment, and the difference was statistically significant (P<0.05) (Table-I).

**Table-I:** Comparison of levels of PCT and CRP between the three groups before treatment (mean±SD).

*Group*	*N*	*PCT(μg/L)*	*CRP(mg/L)*
Infection group	98	2.52±2.89	73.81±18.27
Non-infection group	66	0.17±0.07	7.91±3.01
Normal control group	50	0.02±0.01	3.53±1.26
F		13.324	10.183
p		<0.05	<0.05

### Comparison of PCT and CRP levels between the infection group and the non-infection group before and after treatment

The levels of PCT and CRP of the infection group after treatment were much lower than those before treatment, and there was an obvious difference (P<0.05); the PCT and CRP levels of the non-infection group indicated no remarkable difference before and after treatment (P>0.05); the level of PCT of the infection group was higher than that of the non-infection group after treatment (P<0.05), and the CRP level of the infection group was not remarkably different with that of the non-infection group (P>0.05) (Table-II).

**Table-II:** Comparison of the levels of PCT and CRP between the infection group and non infection group before and after treatment (mean±SD).

*Group*		*PCT(μg/L)*	*CRP(mg/L)*
Infection group	Before	2.52±2.89	73.81±18.27
	After	1.97±0.13*^#^	7.34±2.66*
Non-infection group	Before	0.17±0.07	7.91±3.01
	After	0.23±0.06	7.27±2.43

***Note:***

* indicated P<0.05 compared to before treatment;

# indicated P<0.05 compared to the non-infection group after treatment.

### The diagnosis of COPD in combination with bacterial infection based on PCT and CRP levels

COPD in combination with bacterial infection was diagnosed by PCT alone, CRP alone and PCT in combination with CRP. The results demonstrated that, the diagnostic indexes of the combination detection were superior to those of single detection (Table-III).

**Table-III:** Efficiency analysis of diagnosing COPD in combination with PCT and CRP levels (%).

*Index*	*N*	*Specificity*	*Sensitivity*	*Missed diagnosis rate*	*Misdiagnosis rate*
PCT	98	86(87.8)	68(69.4)	30(30.6)	11(11.2)
CRP	98	65(66.3)	91(92.9)	7(7.1)	33(33.7)
PCT in combination with CRP	98	88(89.8)	89(90.8)	5(5.1)	10(10.2)

## DISCUSSION

The incidence of COPD is 9% to 10% among patients over 40 years old, and moreover the morbidity and mortality rate of COPD remain high, which severely threatens the lives of patients. It has been found that, about 25% to 50% of COPD cases are induced by bacterial infection.^10^ Previously, clinical doctors determine bacterial infection according to experience or the changes of WBC and ESR; however, error diagnosis is easy to happen due to the low specificity and sensitivity, which can bring severe adverse impacts to the subsequent treatment of patients.^11^ PCT as a marker for bacterial infection has attracted more and more attentions in recent years. PCT in combination with CRP is valuable in the diagnosis of COPD in combination with bacterial infection and reasonable use of antibacterial drugs. Calcitonin (PCT) is a kind of polypeptide hormone extracted from the culture solution of thyroid tumor at the earliest. Therefore, it is a serological marker for thyroid tumor. PCT is the precursor of CT, has an extremely low level in the serum of normal people. But the level of PCT is high in the serums of patients with systemic inflammatory reactive syndrome, acute and chronic pneumonia. Compared to WBC, interleukin (IL)-6, tumor necrosis factor (TNF)-2, CRP and soluble selectin, PCT is a quite sensitive serologic marker, which is free from the influence of hormone level in human body; moreover, it is beneficial to detection because of its high stability in vivo and vitro.^12^,^13^ The research results suggested that, the PCT level of the infection group was remarkably different with that of the normal control group before treatment (P<0.05); the serum PCT level of the infection group and the non-infection group was significantly different with that of the normal control group (P<0.05); after treatment, the PCT level of the infection group and non-infection group had an obvious difference (P<0.05). The above findings suggested that, PCT was sensitive to bacterial infection reaction, and its level in the body of healthy people was so low that almost could not be detected. Thus PCT is an important marker for bacterial infection and also an important index for the determination of the category and activity of inflammation.

CRP as an acute phase protein indicates a significantly higher level in the early stage of inflammation. Besides bacterial infection, acute rejection reaction and surgery can also induce the increased level of CRP, suggesting the lack of specificity to infection; after the termination of inflammatory stimulus, the synthesis of CRP in the liver can still last for several days.^14^,^15^ The results of this study suggested that, the CRP level of the infection group and non-infection group had a remarkable difference (P<0.05); the CRP level of the infection group and non-infection group was significantly different with that of the normal control group before and after treatment (P<0.05); the level of CRP of the infection group and non-infection group had significant difference after treatment (P>0.05). It could be concluded that, CRP was unable to help distinguish the degree of bacterial infection, especially in the late stage. Through comparing the PCT and CRP levels of 169 patients without infection and 200 patients with infection, Zhang Yanping et al. found that,^16^ the CRP and PCT levels of the infection group were much higher than those of the non-infection group before treatment ((73.16±8.35) mg/L vs (7.89±2.43) mg/L); ((2.47±0.52) μg/L vs (0.19±0.07) μg/L) and (11.31±3.63) mg/L vs (7.23±1.87) mg/L; (1.98±0.45) μg/L vs (0.18±0.06) μg/L) after treatment. The research results of this study were consistent with the above conclusions, suggesting the results of this study were scientific and referable.

## CONCLUSION

PCT level in combination with CRP level is a sensitive and specific index for determining the existence of bacterial infection in AECOPD patients. The detection method is featured by simple operation and high practicability. The detection of PCT level in the course of disease is beneficial to clinical application. Therefore, all AECOPD patients are suggested for undergoing serum PCT and CRP levels detection before treatment to help the diagnosis of bacterial infection and guide the reasonable use of antibacterial drugs.

### Authors’ Contribution

***YYL:*** Study design, data collection and analysis.

***LLX, SZX & KSL:*** Manuscript preparation, drafting and revising.

***YYL & KSL:*** Review and final approval of manuscript.
